# Severe Extra-Cerebral Anticoagulant-Related Bleeding in Intensive Care Unit: A Retrospective Study From 2000 to 2013

**DOI:** 10.1097/MD.0000000000002161

**Published:** 2015-10-30

**Authors:** M Hauguel, Py Boelle, C Pichereau, S Bourcier, N Bigé, JL Baudel, E Maury, B Guidet, H Ait-Oufella

**Affiliations:** From the AP-HP, Hôpital Saint-Antoine, Service de Réanimation Médicale (MH, CP, SB, NB, JB, EM, BG, HA-O), Inserm U970, Paris Research Cardiovascular Center (HA-O), AP-HP, Hôpital Saint-Antoine, Service de Santé Publique (PB), Université Pierre et Marie Curie-Paris 6 (PB, CP, EM, BG, HA-O), and UMRS_1136, Paris, France (PB, EM, BG).

## Abstract

Bleeding is the most frequent complication of anticoagulant therapy, responsible for a number of hospitalizations or deaths. However, studies describing the management and prognosis factors of extra-cerebral anticoagulant-related bleedings in intensive care unit (ICU) are lacking.

Retrospective observational study in an 18-bed ICU in a tertiary teaching hospital. From January 2000 to December 2013, all consecutive patients, older than 18 years, admitted for severe anticoagulant-related bleeding (SAB) except intracerebral site were included.

A total of 100 patients were included, the mean age was 77 ± 11 years and 62% were women. SAB incidence in ICU doubled over 10 years (*P* = 0.03). In ICU, the average length of stay was 5 ± 6 days and mortality was 30%. Nonsurviving patients had a higher SAPS II (78 ± 24 vs 53 ± 24, *P* < 0.0001), a higher SOFA (9.0 ± 3.6 vs 4.7 ± 3.4, *P* < 0.0001) and received more frequently support therapy such as mechanical ventilation (87% vs 16%, *P* < 0.0001) and vasopressors (90% vs 27%, *P* < 0.0001). The volume of blood-derived products transfused was more important in nonsurvivors mainly during the first 24 hours of resuscitation. Rapid anticoagulant reversal therapy was associated with better prognosis (ICU survivors 66% vs 39%, Fisher test *P* = 0.04). Anterior abdominal wall was identified as a frequent site of bleeding (22%) due to epigastric artery injury during subcutaneous injection of heparin and was associated with a large mortality (55%).

Extra-cerebral SAB is a life-threatening complication that requires rapid resuscitation and anticoagulant reversal therapy. Injection of heparin should be done carefully in the subcutaneous tissue thereby avoiding artery injury.

## INTRODUCTION

Anticoagulant therapy is the cornerstone of treatment and prevention of thrombosis in numerous clinical settings.^1^ Numerous studies have reported that anticoagulant therapy reduces the risk of thromboembolic complications in clinical conditions such as atrial fibrillation, mechanical heart valves, deep vein thrombosis, pulmonary embolism, and cardiogenic stroke. Bleeding is the most frequent and life-threatening complication of anticoagulant therapy,^2^ responsible for a number of hospitalizations or deaths.^3,4^ In a large cohort of patients in the United States, warfarin was identified as the first drug responsible for hospitalizations related to adverse drug events (33% of the cases).^5^ Annual bleeding rates range from 0% to 4.8% for fatal bleeding and range from 2.4% to 8.1% for major bleedings requiring hospitalization.^6^ The major determinants of anticoagulant-induced bleeding are the intensity of the anticoagulant effect, patient characteristics, the concomitant use of drugs that interfere with hemostasis and the length of therapy.^7^ Despite the frequency and the severity of anticoagulant-related haemorrhages, studies reporting the management of extra-cerebral bleeding in intensive unit care are lacking.^8^ The aim of this study was to describe epidemiological and clinical characteristics of patients admitted in intensive care unit (ICU) for severe extra-cerebral anticoagulant-related bleeding and to identify prognosis factors.

## METHODS

We conducted a retrospective observational study in an 18-bed ICU in a tertiary teaching hospital. From January 2000 to December 2013, all consecutive patients, older than 18 years, admitted for anticoagulant-related bleeding were included. Patients were identified by querying the electronic health records with the following keywords (in French): “bleeding,” “anticoagulant,” “iatrogenic,” “heparin,” “vitamin K antagonist” (VKA), “complication,” and “hemorrhage.” Anticoagulant overdose was defined for VKA as an International Normalized Ratio (INR) >3, for low-molecular weight heparin (LMWH) as anti-Xa activity >1 at H6 from latest heparin injection, and for unfractionated heparin (UFH) as anti-Xa activity >0.6 or activated partial thromboplastin time (aPTT) >3 at H6 from latest heparin injection. Novel oral anticoagulant (NOAC) overdoses were not assessed in routine exams in our hospital. Patients with intracranial bleeding were not included in this study because we aimed at describing severe bleeding responsible for hemorrhagic shock with organ failure. The management and the outcome of brain hemorrhage are not dependent on blood volume lost, and prognosis factors could not be applied for extra-cerebral bleeding.^9^ To analyze the changes of severe anticoagulant-related bleeding (SAB) incidence, we compared 2-year periods between 2000 and 2013. General characteristics of the patients were recorded: demographic, biological data, diagnoses, severity of illness evaluated by the Sequential Organ Failure Assessment (SOFA) score^10^ (at admission and at H24) and Simplified Acute Physiology Score II (SAPS II)^11^ and therapeutic management. The origin of bleeding was adjudicated by 2 independent reviewers to distinguish spontaneous or iatrogenic bleeding based on the screening of potential medical interventions (injection, puncture, and biopsy) before hemorrhage and CT-scan imaging.

### Statistical Analysis

Patients’ characteristics were summarized as mean ± standard deviation, median (25–75th percentiles) for skewed distributions, and percentages as appropriate. Comparisons were assessed using the Fisher exact test for categorical variables because of small sample size. We used the Student *t*-test or the Mann–Whitney *U* test for continuous variables according to their distribution. *P* value <0.05 was considered statistically significant. Finally, we used logistic regression for multivariable analysis. All statistical analyses were performed using the R software (v 2.12.0; http://cran.r-project.org).

This is an observational retrospective study with an anonymous analysis. All patients and families were informed through the admission leaflet that anonymous data could be used for academic research. For these reasons, an ethical committee approval was not required.

## RESULTS

### Studied Population

Between January 2000 and December 2013, 12,214 patients were admitted in our ICU and 122 patients were admitted for an SAB. Twenty-two patients were excluded, 3 patients because of missing data and 19 due to intracerebral bleeding leaving 100 patients for study. SAB incidence significantly increased from 0.5% in 2000 to 2001 period to 1.1% during 2012 to 2013 period (*P* = 0.03) but global severe-bleeding incidence (with or without anticoagulant therapy) did not change between both periods (4.6% vs 5.0%, *P* = NS). Overall, patients received anticoagulant therapy for venous thromboembolism disease (38%), atrial fibrillation (46%), heart valve disease (11%), and acute coronary syndrome (2%). All patients had a curative anticoagulant regimen. 70% of the patients received VKA, 60% received heparin (23% UFH, 37% LMWH), and 31% received both during a bridging therapy. Finally, 5 patients had fondaparinux and 1 NOAC. Anticoagulant dose was supratherapeutic in 60% of the cases at ICU admission.

Characteristics of the patients are summarized in Table 1. The mean age was 77 ± 11 years, mostly women. The average length of stay in the ICU was 5 ± 6 days and 31 ± 33 days in hospital. ICU-mortality was 30% and overall in hospital-mortality was 34% (median time of death 4.0 [2.0–5.7] days). Patients dying in ICU had a higher SAPS II, a higher SOFA (at admission as well as at 24 hours) and received more frequently support therapy such as mechanical ventilation (87% vs 16%, *P* < 0.0001) and vasopressors (90% vs 27%, *P* < 0.0001). However, comorbidities prevalence (hypertension, chronic heart failure, chronic renal failure, cirrhosis, and diabetes) was not different between ICU survivors and nonsurvivors.

**TABLE 1:**
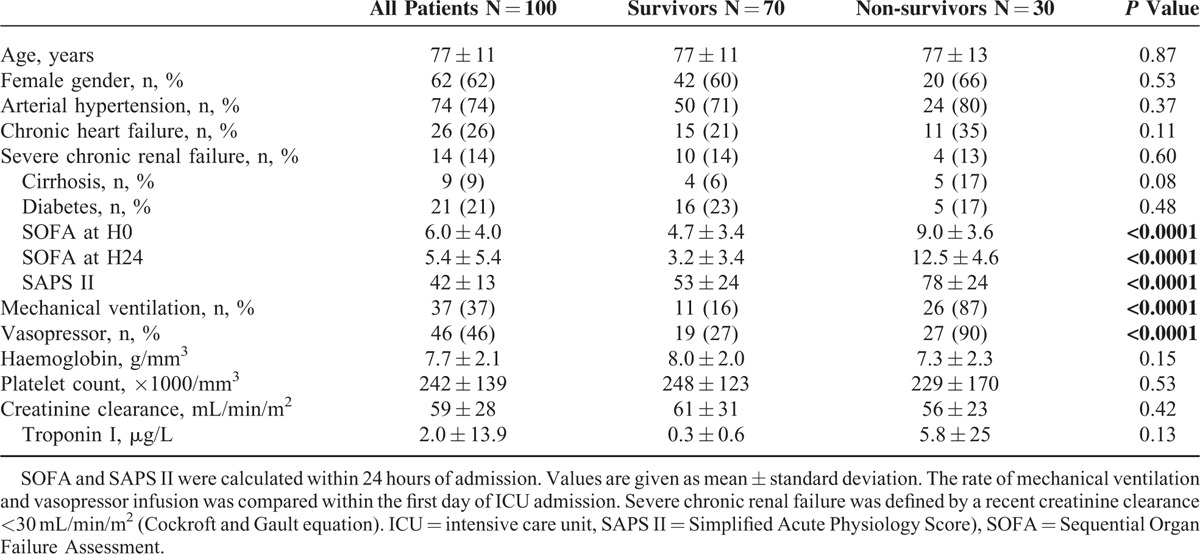
Clinical and Biological Characteristics of Studied Population According to ICU Outcome

### Sites of Bleeding

**FIGURE 1:**
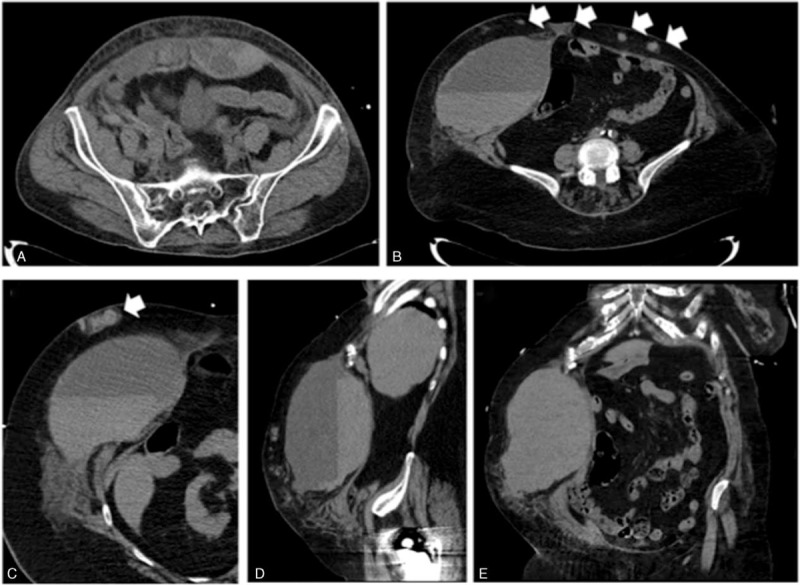
Computed tomography (CT)-scan imaging of bleeding in the anterior abdominal wall. (A) Bleeding in the rectus abdominis muscle. (B, C) Horizontal sections showing an important bleeding in the right rectus abdominis muscle associated with several subcutaneous hematomas (white arrows), sagittal section (D), and coronal section (E).

The major sites of bleeding were the gastro-intestinal tract (31%), the retroperitoneum (29%), and the anterior abdominal wall within the rectus abdominis muscle (Fig. 1A–E) (22%). Bleeding was observed in the thorax in only 6% of the cases and rarely in other tissues (calf or thigh).

Bleeding was mainly spontaneous when located in the gut (97%) and in the retroperitoneum (79%). However, regarding anterior abdominal wall area, spontaneous bleeding was rare (9%, Fisher test, *P* < 0.0001) and occurred frequently after an intraabdominal injection of heparin (Fig. 1A, B). Indeed, we identified that abdominal pain and symptoms occurred after injection of anticoagulant. CT-scan confirmed that anterior wall bleeding originated from tissue anticoagulant injection leading to epigastric artery injury (Fig. 1B). In addition, we observed numerous abdominal subcutaneous hematomas in patients who secondary had severe bleeding in the anterior wall area (Fig. 1B, C).

### Bleeding Management

At admission, hemoglobin level, platelet count, or kidney functions were not different between ICU survivors and nonsurvivors (Table 1). Nonsurvivors received more red blood cells, platelets, and fresh frozen plasma units than survivors. Differences were significant for products that had been administrated within the first 24 hours of ICU admission (Table 2). Among VKA-treated patients (n = 39), anticoagulant reversal was performed in all the patients (100%): vitamin K in 97% of the patients, prothrombin complex concentrate (PCC) in 92%, and both treatment in 87% of the patients. Among heparin-treated patients (n = 29), 65% of them received anticoagulant reversal therapy, either by protamine (10/29, 34%) or by fresh frozen plasma (9/29, 31%). Seventy percent of the patients with VKA-treatment combined with LMWH/UFH bridging (n = 31) were treated with an antidote. Vitamin K was administered in 17 patients (55%), PCC and protamine in 7 patients (22%), and FFP in 1 patient (3%). We observed that rapid anticoagulant reversal treatment, that is, <12 hours between symptoms onset and anticoagulant reversal, was associated with better prognosis (ICU survival 66% vs 39%, *P* = 0.04). The association between timing of antidote administration and prognosis remained significant after adjustment on SOFA score (OR 3.86 95%CI [1.03–14.43], *P* = 0.04). VKA-related bleeding was associated with a lower rate of mortality (*P* = 0.004). In contrast, there was no relation between ICU outcome and other anticoagulant drugs (UFH, LWMH, and NOAC). Furthermore, ICU mortality was not associated with anticoagulant overdose (18/60 in overdose group vs 12/40 in nonoverdose group, *P* = NS).

**TABLE 2:**
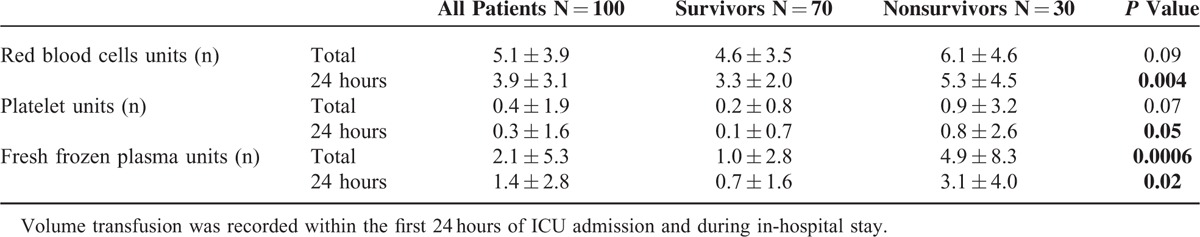
Volume of Transfusion of Red Blood Cells, Platelet, and Fresh Frozen Plasma Unit According to Intensive Care Unit (ICU) Outcome

A hemostatic procedure including surgery, endoscopy, or endovascular embolization was performed in 44% of the cases. For gastrointestinal bleedings, most of the patients underwent endoscopy (77%). Hemostatic procedure was performed in 10 patients (34%) admitted for hemoretroperitoneum (surgery n = 1, endovascular embolization n = 9) and in 6 patients (27%) admitted for anterior abdominal wall hemorrhage (surgery n = 3, endovascular embolization n = 3).

### Anterior Abdominal Wall Bleeding

We observed that ICU mortality was high among patients with anterior abdominal wall bleeding (55% vs 23%, *P* < 0.05). To investigate this specific poor outcome, we compared patients with anterior abdominal wall bleeding to other patients (Table 3). Patients with anterior abdominal wall bleeding were mostly women with higher body mass index receiving more frequently heparin and fondaparinux. Patients with anterior abdominal wall bleeding had a higher SAPS II, a higher SOFA and received more frequently support therapy such as mechanical ventilation and vasopressors. There was no difference of anticoagulant reversal therapy between groups in term of rate of antagonization and time between symptoms and antidote administration.

**TABLE 3:**
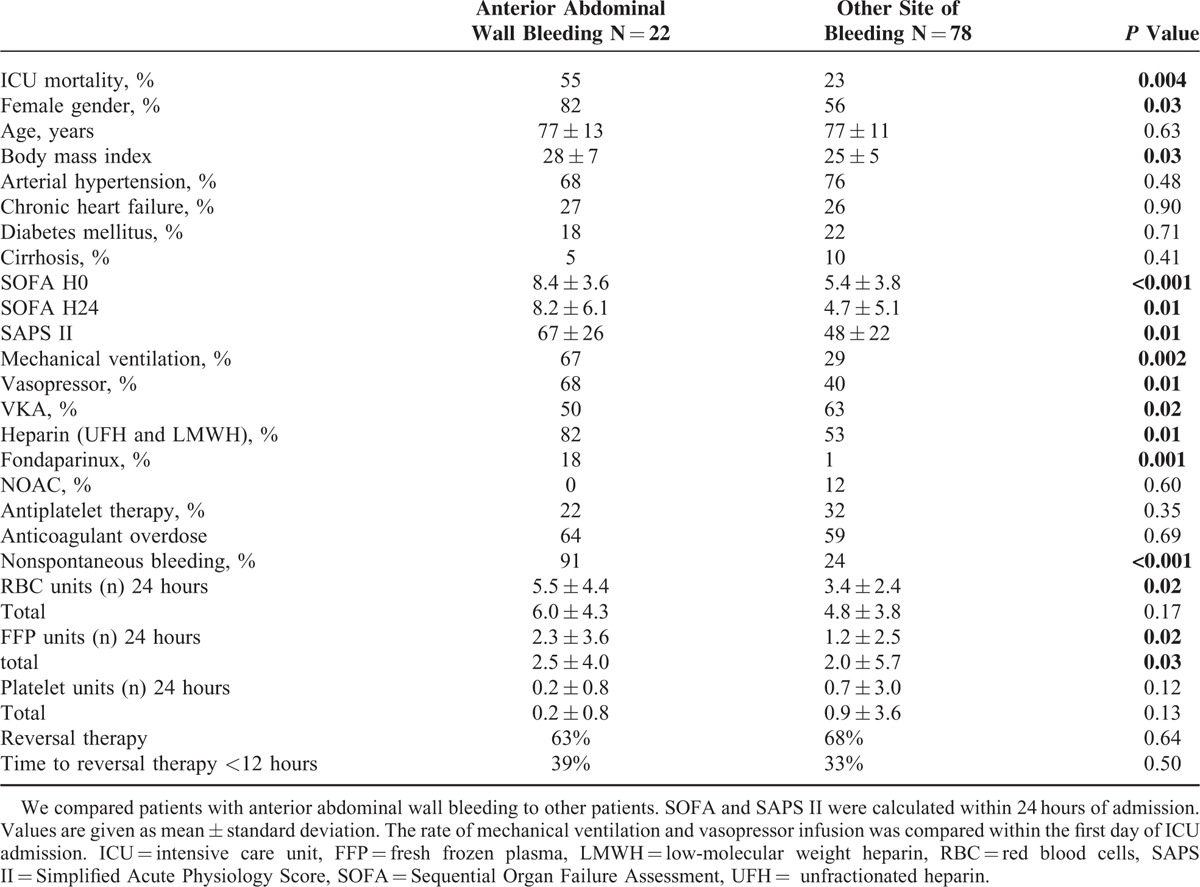
Clinical and Biological Characteristics of Patients According to the Site of Bleeding

Using a logistic regression, we adjusted the analysis of anterior abdominal wall bleeding for parameters related to patients’ condition and to ICU management (rapid vs delayed anticoagulant reversal treatment). We found an increased mortality in patients that received delayed antidote (ie, >12 hours) (OR = 3.4, 95%CI (1.1–10.4), *P* = 0.03). Anterior abdominal wall bleeding was also associated with increased mortality (OR = 4.0, 95%CI [1.5–10.8], *P* = 0.003). Adjustment on anticoagulant therapy indications and on the number of comorbidities did not change the strength of the associations, although some were less significant (Table 4).

**TABLE 4:**
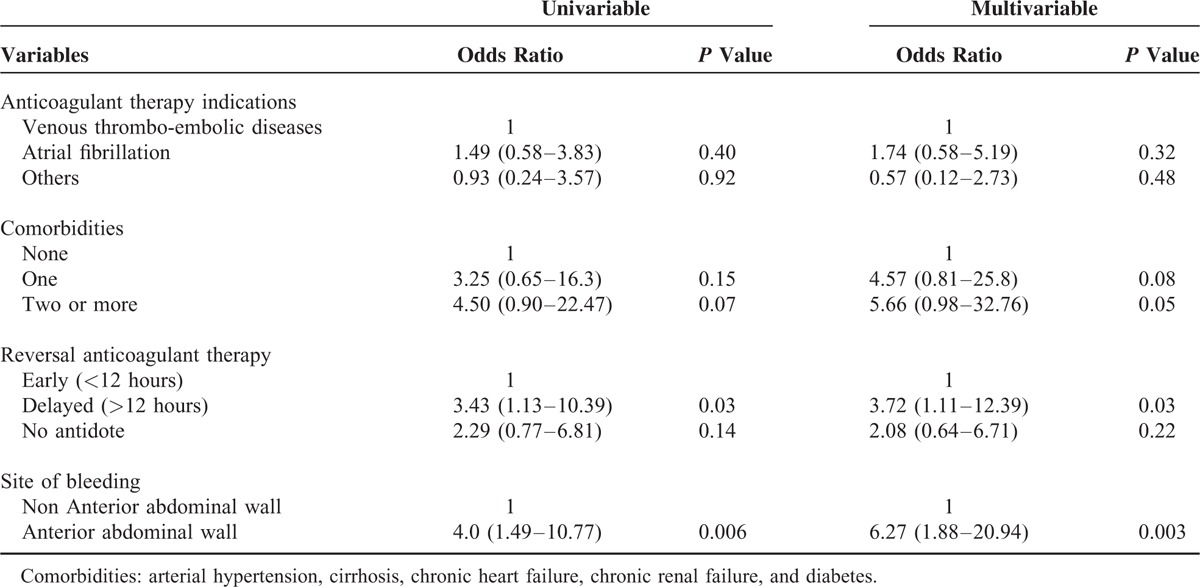
Multivariable Logistic Regression Analysis of Risk Factors for Intensive Care Unit (ICU) Mortality

## DISCUSSION

Between 2000 and 2013, we observed a 2-fold increase of extra-cerebral SAB incidence and pointed out the severity of this specific anticoagulant-associated adverse event, with frequent multiorgan failure requiring organ support therapy leading to high ICU mortality. We found that anterior abdominal wall was a frequent site of bleeding complication associated with a pejorative prognosis. We reported a significant relation between the time to anticoagulant reversal therapy and the outcome.

SAB incidence significantly increased during the 14-year period. As all patient admissions have been entered in a computerized system since 2000, no selection bias can explain this increase. Full text searching for hemorrhagic manifestations and anticoagulant therapy in the electronic health records allowed maximizing the recall of our query. The increase of SAB incidence was probably due to patients aging who have more frequent cardiovascular diseases requiring anticoagulant therapy.^12,13^ In agreement, the age of patients admitted in our ICU increased from 59 to 65-year old (median) between 2000 and 2013. Finally, the recent extension of the anticoagulant therapy indications for atrial fibrillation, that is, CHA_2_DS_2_VASc score ≥1, could also explain our epidemiological observation.^14^

In our study, patients admitted for extra-cerebral SAB were mostly elderly women. In a large cohort of patients with deep venous thrombus, White et al^15^ previously identified the age (over 65-year old) and the female sex as risk factors of bleeding. As compared to younger patients, elderly patients have about a 5-fold higher incidence of major and fatal bleeding.^16^ In critically ill context, Serghini et al^8^ also reported a predominance of women. Comorbidities prevalence was not statistically different between ICU survivors and nonsurvivors. However, we observed an increase of chronic heart failure and cirrhosis prevalence among nonsurvivors that did not reach statistical significance probably because of limited sample size and in fine low statistical power.

Regarding patient's management, the volume of blood-derived products transfusion was more important in nonsurvivors mainly during the first 24 hours of ICU admission suggesting a more active bleeding in this group. In trauma context, del Junco et al^17^ reported similar results. The relationship between volume transfusion and the outcome was only significant when the authors focused on volume transfusion within the first 4 hours of resuscitation. Hemoglobin level at admission was not different between survivors and nonsurvivors, which demonstrated the lack of sensitivity of this biological parameter to accurately evaluate the severity confirming numerous studies in the context of gastrointestinal bleeding.^18^ Reversal of anticoagulant therapy has been done in 100% of the VKA-treated patients and 65% of the heparin-treated patients. This result is better than the current clinical practice reported by Desmettre et al^19^ in a multicenter prospective analysis. Indeed, an appropriate administration of Prothrombin complex concentrate for reversal of VKA was performed in only 26% of the cases. It is well admitted that anticoagulant overdose was associated with an increased risk of bleeding^7^ but the relationship between drug overdose and prognosis among anticoagulant-treated patients with active bleeding remains unknown. In our cohort, we did not observe any association between ICU outcome and anticoagulant overdose. This finding has to be interpreted with caution but could be explained by the fact that reversal therapy was administrated in the majority of the patients and allowed a rapid normalization of blood coagulation.

We reported a significant relation between the time to anticoagulant reversal therapy and the outcome independently to bleeding severity. In addition, the timing of bleeding source (surgical or endovascular) control is also of paramount importance. However, we did not analyze this aspect of therapeutic management because the number of hemostatic procedures (surgery n = 4, endovascular embolization n = 12) was no sufficient to perform informative statistical analysis.

A peculiar feature of our patient's sample was the high number of anterior abdominal wall bleedings associated with a poor prognosis. The association between anterior abdominal wall bleeding and mortality remained significant after adjustment on comorbidities and timing of reversal therapy. The severity of patients with anterior abdominal wall bleeding was already higher at admission with more frequent organ support therapy and higher volume transfusion of blood-derived products suggesting a more active hemorrhage. CT-scan analysis confirmed active bleeding originating from epigastric arteries. Imaging and clinical history suggested that the hemorrhagic accident was due to direct vascular injury during injection of heparin. Physicians and nurses should be aware of this serious adverse event and heparin injection should be done carefully in the subcutaneous tissue. The thigh or the deltoid area is an alternative site of injection that could limit adverse events. In noncritically ill context, Rectus Sheath bleedings have been described by others. Between 1992 and 2002, Cherry and Mueller^20^ retrospectively recorded 126 cases. Most the patients were women (64%) receiving anticoagulant therapy (69%). More recently, Sheth et al^21^ retrospectively described the characteristics of 115 patients admitted for a Rectus Sheath Hematoma. Most of the patients received anticoagulant therapy (77%), mainly heparin injection (51%).

## LIMITATIONS

Most anticoagulant therapies included VKA and/or heparin, so that our findings may not be relevant for NOAC, the use of which has been increasing in Europe since 2012. Therefore, the outcome of severe bleeding complication in patients could change overtime, as treatment with NOAC implies less direct monitoring and a specific antidote is lacking.^3^

Finally, we performed a retrospective study and it is difficult to definitely exclude some biases. To limit recruitment bias, we included patients from 2000, when the electronic health recording system was fully available in our hospital.

## CONCLUSION

Extra-cerebral SAB admitted in ICU represented a severe drug event with multiorgan failure requiring frequent support therapy and was associated with high mortality. Injection of heparin should be done carefully in the subcutaneous tissue to avoid artery injury.
